# The regulation of miR-320a/XBP1 axis through LINC00963 for endoplasmic reticulum stress and autophagy in diffuse large B-cell lymphoma

**DOI:** 10.1186/s12935-021-01992-y

**Published:** 2021-06-10

**Authors:** Yuying Cui, Hui Xu, Yu Yang, Dongmei Zhao, Yu Wen, Chao Lv, Hongbin Qiu, Chennan Wang

**Affiliations:** 1 School of Basic Medicine, Jiamusi University, Jiamusi, 154007 Heilongjiang China; 2 School of Public Health, Jiamusi University, Jiamusi, 154007 Heilongjiang China; 3 School of Clinical Medical, Jiamusi University, Jiamusi, 154007 Heilongjiang China

**Keywords:** Bioinformatics, LINC00963, miR-320a, XBP1, ERs, Apoptosis, Autophagy

## Abstract

**Background:**

This study incorporates fundamental research referring to considerable amounts of gene-sequencing data and bioinformatics tools to analyze the pathological mechanisms of diffuse large B-cell lymphoma (DLBCL).

**Methods:**

A lncRNA-miRNA-mRNA ceRNA network of DLBCL was constructed through database analysis combining GTEx and TCGA. qPCR was used to detect the expression of LINC00963 and miR-320a in DLBCL cell lines. After LINC00963 or miR-320a overexpression in vitro, western blot was performed to assess the protein levels of UPR sensors (GRP78, p-IRE1, IRE1, active ATF6, ATF4 and XBP1), along with apoptosis markers (Bcl-2, Bax, caspase 3) and autophagy indicators (Beclin1, LC3II, LC3I and p62). Additionally, the expression of LC3 was analyzed through immunofluorescence (IF) assay.

**Results:**

Following LINC00963 overexpression in vitro, SUDHL4 cell line showed a marked increase in the level of UPR-related GRP78, p-IRE1 and spliced XBP-1/XBP-1(s), apoptosis-related Bax and cleaved caspase 3, as well as autophagy-related Beclin1 and LC3II, whereas miR-320a mimic greatly diminished the effects of LINC00963 overexpression. Moreover, LINC00963 targeted miR-320a while miR-320a bound to the 3’UTR of XBP1. It was also found that LINC00963 overexpression resulted in significantly delayed tumor growth in a xenograft model of DLBCL.

**Conclusion:**

Mechanistically, LINC00963/miR-320a regulated XBP1-apoptosis pathway and autophagy, implying the therapeutic potential of this pathway for selective targeting. The data presented here illustrated the mechanism of LINC00963/miR-320a/XBP1 in DLBCL for the first time.

**Supplementary Information:**

The online version contains supplementary material available at 10.1186/s12935-021-01992-y.

## Introduction

Diffuse large B-cell lymphoma (DLBCL) accounts for 30–40% percent of non-Hodgkin’s lymphoma (NHL) and is the most common form of NHL. DLBCL is characterized by rapid progression, and untreated patients with this disease may only have less than a year to live [1]. The treatment of non-Hodgkin’s lymphomas with superior vena cava obstruction, tumor lysis syndrome could be trigged through chemotherapy [2]. Studying and understanding the pathogenesis and development of DLBCL help to identify the potential therapeutic targets and construct new therapeutic strategies. Recent studies have shown that lncRNA accounts for about 80% of the total non-coding RNA (ncRNA) and is involved in various physiological and pathological processes through transcriptional regulation, mRNA processing, spongy action, and nuclear transport [3, 4].

Many studies have demonstrated that LINC00963 as a sponge of miRNA is involved in tumor growth [5–7]. MiR-320a is located on human chromosome 8p21.3 and closely related to disease progression, tumor invasion and metastasis [8–10], and the expression of which could be regulated by different lncRNAs in different diseases [9, 11, 12]. Endoplasmic reticulum (ER) stress and autophagy have both been shown to affect the overall survival in DLBCL patients and the response of DLBCL to chemotherapy [13, 14]. In multiple myeloma, activation of the IRE1-XBP1 ER stress signaling pathway is thought to have a pro-oncogenic effect. However, IRE1-XBP1 expression was found to decrease in diffuse large B-cell lymphoma originating from germinal center B cells (GCB-DLBCL), thus exerting a negative effect on tumor growth. IRE1-XBP1 pathway plays a key role in malignant tumors, which promotes or inhibits tumor growth depending on the type of tumor. XBP1s may be directly involved in pro-apoptotic processes and may thus contribute to the suppression of tumor growth in the lymphoma subtype [15].

Studies have shown that LINC00963 is up-regulated in some cancers, such as breast cancer and liver cancer, and plays a promoting role in cancer progression  [16, 17]. However, database analysis in the present study based on GTEx and TCGA databases found that LINC00963 was down-regulated in GCB-DLBCL, and its low expression was significantly associated with poor prognosis. Therefore, its role in DLBCL is need of an in-depth investigation.

In this present study, we wonder to know the role of LINC00963 in diffuse large B-cell lymphoma and conducted in vivo and in vitro experiments to validate how it involves in the pathomechanism of diffuse large B-cell lymphoma based on bioinformatics analysis. LINC00963/miR-320a axis was found to regulate endoplasmic reticulum stress and autophagy in vitro of diffuse large B-cell lymphoma, this regulatory role of which was closely associated with XBP1. Additionally, tumor growth in vivo was suppressed by the induction of LINC00963 overexpression, this effect of which could be mediated by miR-320a/XBP1. In summary, LINC00963/miR-320a/XBP1 could be potential molecular markers and targets for the diagnosis and treatment of diffuse large B-cell lymphoma.

## Method

### Database analysis

The University of California Santa Cruz (UCSC) Genome Browser was used to download the data from GTEx and TCGA databases (normal N = 337, tumor T = 48). The intersection of genes was taken and corrected, and Wilcoxon test (non-parametric test) was used for difference analysis (logFC = 1). The log-rank test and Kaplan-Meier method were applied to the analysis of the correlation between lncRNA expression and overall survival rate. StarBase 3.0 was searched for potential lncRNA-miRNA interactions. The differentially expressed mRNAs in liver cancer were analyzed using non-parametric test (logFC = 2) through Database of Essential Genes (DEG). Subsequently, candidate mRNAs that may interact with the predicted miRNA were acquired by consulting miRDB, miRTarBase and TargetScan databases. Next, mRNA intersections were established based on the respective mRNA predictions by miRDB, miRTarBase, TargetScan as well as DEG. Cytoscape 3.7.2 was used to construct the lncRNA-miRNA-mRNA competitive endogenous RNA (ceRNA) network. At last, GO enrichment analysis was performed to evaluate the potential functions of RNA molecules in coexpression networks.

### Cell culture

Non-cancerous human B lymphocytes GM12878 and DLBCL cell lines SUDHL4, OCI-Ly1, HBL1 and OCI-Ly3 were obtained from American Type Culture Collection (ATCC; Manassas, VA, USA). GM12878 cells were cultured in RPMI 1640 medium (HyClone, USA) containing GlutaMAX Supplement (Thermo Fisher Scientific, USA), 15% fetal bovine serum (FBS, Sigma-Aldrich, USA) and 1% penicillin/streptomycin (Invitrogen, USA). SUDHL4 and OCI-Ly1were grown in DMEM medium with 10% FBS and 1% penicillin/streptomycin. HBL1 and OCI-Ly3 were cultured in Iscove’s modified Dulbecco’s medium (SIGMA, America) with 20% human serum medium and 1% penicillin/streptomycin.

### qPCR

Total RNA of cells was extracted by TRIzol-based method. After GM12878, DLBCL, SUDHL4, OCI-Ly1, HBL1 and OCI-Ly3 cellswere centrifuged at 48,000 r/min for 5 min, the supernatant was discarded, and the cells were incubated with 1 ml of TRIzol reagent followed by 200 µl of chloroform respectively for 15 min. Next, the cells were centrifuged at 12,000r/min at 4 °C for 10min. The RNA was reverse transcribed into cDNA using a cDNA reverse transcription kit (TAKARA, Japan) according to the manufacturer’ instructions. The cDNA was amplified using SYBR PremixEx TaqII kit (TAKARA, Japan). GADPH was used as internal control. The 2^−∆∆Ct^ method was used to calculate the relative expression of LINC00963, miR-320a, XBP1(s) mRNA and XBP1(u) mRNA.

### Plasmid transfection

Plasmids overexpressing LINC00963 and the empty vector were purchased from Genechem (Shanghai, China). MiR-320a mimic, miR-320a inhibitor and the respective negative controls were designed by GenePharma (Shanghai, China). Lipotransfectamine 3000 was used to transfect SUDHL4 cells (Thermo Fisher Scientific, USA). After transfection of 48 h, cells were collected to perform the following experiments.

### CCK8 assay

SUDHL4 cells were seeded into a 96-well plate in a concentration of 100 µL/well (5*10^4^ cells) and were observed at 24, 48 and 72 h after transfection. 10 µL of CCK-8 reagent was added to each well and incubated for 4 h. The absorbance (OD) at 450 nm was determined by a microplate microscope for 3 times. 5 wells were set for each group in this assay.

### Western blot

After treatment in different groups, SUDHL4 cells were collected, lysed on ice with RIPA buffer for 30 min and centrifuged at 12,000 g for 20 min to discard the supernatant. The cell lysate underwent SDS-PAGE electrophoresis and then transferred onto a PVDF membrane. Afterwards, the protein bands were blocked with non-fat milk powder dissolved in PBS (5% wt/vol) for 1 h and incubated with the primary antibodies against GRP78, p-IRE1α, IRE1α, active ATF6, ATF4, XBP1(s), XBP1(u), Bcl-2, Bax, caspase 3, Beclin1, LC3II, LC3I,p62 and GAPDH (Abcam, England) respectively a 4 °C overnight. Then, protein bands were incubated with HRP-conjugated secondary antibodies (Abcam, England) for 2 h at room temperature. GAPDH was used to be as an internal reference. The gray value was semi-quantified via Image J software.

### Luciferase reporter assay

The pmirGLO dual-luciferase vector containing LINC00963 sequences (LINC00963 WT/MUT) or XBP1 sequences (XBP1 WT/MUT) was co-transfected into SUDHL4 cells with miR-320a mimic or mimic-NC. The fluorescence intensity was detected on a Luciferase Reporter Assay System (Promega, USA).

### Flow cytometry

SUDHL4 cellswere collected after 48 h of transfection. 1*10^4^ cells were resuspended in 195 µl of Annexin V-FITC binding solution with added 5 µl of Annexin V- FITC (Beyotime, Nanjing, China). Then, 10 µl of propidium iodide staining solution was added to incubate the cells for 20 min. Lastly, the apoptotic cells were detected by a flow cytometer.

### Immunofluorescent (IF) staining

SUDHL4 cells were fixed using 4% paraformaldehyde at 4 °C for 15 min after plasmid transfection, followed by permeabilization with 0.2% Triton X-100 at room temperature for 15 min. Primary antibody against LC3 was added to incubate with the cells at 4 °C overnight. Subsequently, cells were incubated with secondary antibodies for 90 min. DAPI was used to stain the nuclei. The cells were observed under an inverted microscope (Olympus, Tokyo, Japan).

### Nude mouse xenograft model

SUDHL4 cells were cultured until logarithmic growth stage, digested by trypsin, washed with PBS and resuspended in RPMI 1640 medium. The cells (2.5×10^7^ cells/ml) were subcutaneously injected into the left back of nude mice. The size of the tumor was measured after subcutaneous tumor formation. The study was approved by the ethics committee of Jiamusi University.

### Immunohistochemical assay

Paraffinized sections were routinely dewaxed and underwent antigen retrieval under high pressure. The sections were then incubated with primary antibody against Ki-67 at 4 ℃ overnight (CST, USA). After being washed three times using PBS, the sections were incubated with the secondary antibody at room temperature for 20 min. Then, streptavidin-peroxidase was used to stain the sections for 20 min. Following PBS washing, DAB was added to develop color. At last, the sections were observed under a microscope.

### Statistical analysis

Experimental data were shown as mean ± SD and analyzed using one-way analysis of variance followed by turkey’s test. The statistical analysis was performed using Prism software. P < 0.05 was considered statistically significant.

## Results

### lncRNA-miRNA-mRNA ceRNA network in DLBCL was constructed by database analysis combining GTEx and TCGA

**Fig. 1:**
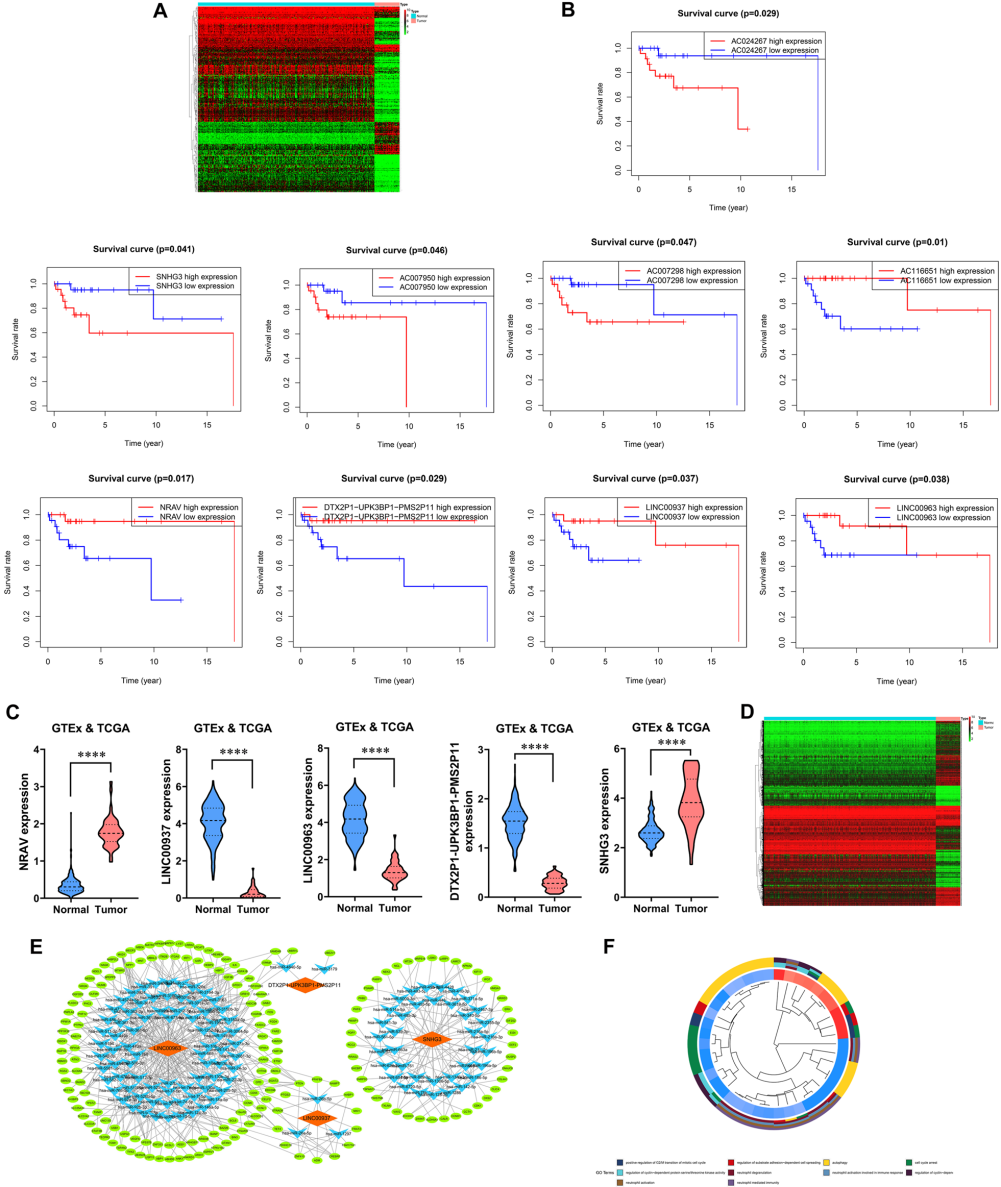
**A** the analysis of heap map for DLBCL data of UCSC database (normal N  = 337, DLBCL T  = 48). **B** The Kaplan–Meier survival analysis about LncRNA in DLBCL patients. **C** The expression of lnc RNA containing NRAV,LINC00937, LINC00963, DTX2P1-UPK3BP1-PMS2P11 and SNHG3 in DLBCL tumor tissue and normal tissue. **D** Heap map about mRNAs differently expressed in DLBCL. **E** ceRNA network of lncRNA-miRNA-mRNA. **F** Functional enrichment analysis of co-expressed genes (Additional File 1)

USCS database was searched for differentially expressed lncRNAs in DLBCL, and a heap map analysis was performed, as shown in Fig. 1A. The log-rank test in Kaplan-Meier survival analysis was used to evaluate the correlation between lncRNA expression and the overall survival (Fig. 1B), where 9 lncRNAs showed significant correlation (P < 0.05). Through lncRNA screening, 5 lncRNAs (NRAV, LINC00937, LINC00963, DTX2P1-UPK3BP1-PMS2P1 and SNHG3) were identified that share miRNA binding sites, and the expression levels of which in normal tissue and DLBCL are shown in Fig. 1 C. Next step, differentially expressed mRNAs in DLBCL were identified from DEG database for the heap map analysis with Wilcoxon test (Fig. 1D). Based on RNA co-expression and network visualization by Cytoscape, it was found that there might be a targeting correlation among LINC00963/has-miR-320a/XBP1 (Fig. 1E). GO analysis results revealed twelve functional clusters including autophagy and the regulation of cell cycle (Fig. 1F), suggesting the regulatory function of the coexpression network in the process of tumor growth.

### Overexpression of LINC00963 inhibits the proliferation of DLBCL cells

**Fig. 2:**
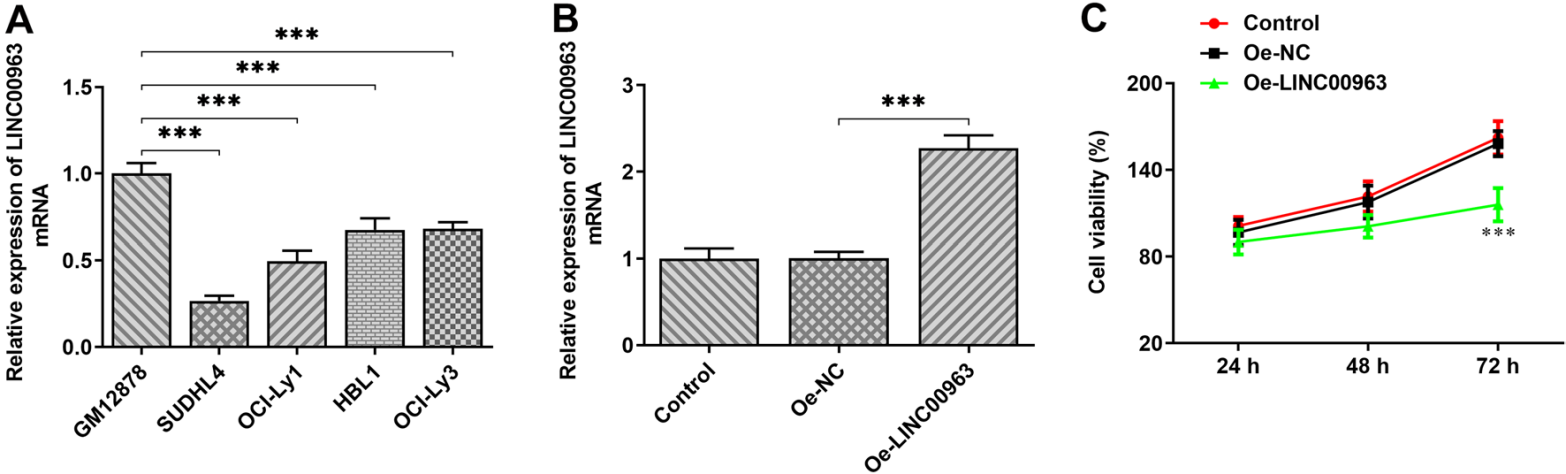
**A** The expression of LINC00963 in different cell lines of DLBCL was detected by qPCR. **B–C** LINC00963 overexpressed plasmid elevated LINC00963 levels and enhanced the proliferation of DLBCL cell

To study possible involvement of LINC00963/has-miR-320a/XBP1, we firstly evaluate the role of LINC00963 following the induction of LINC00963 overexpression. The expression of LINC00963 was detected in DLBCL cell lines and Human B lymphocytes (GM12878) through qPCR. Decreased expression of LINC00963 was observed in DLBCL cell lines compared to GM12878 (Fig. 2A). Furthermore, LINC00963 expression was obviously lower in SUDHL4 cells than that in other DLBCL cells. Therefore, it was reasonable to presume that LINC00963 could play a vital role in DLBCL. Next, LINC00963 overexpression in DLBCL cells was achieved by transfection of the plasmid overexpressing LINC00963, as confirmed by the result of qPCR (Fig. 2B). More importantly, it was further found that LINC00963 overexpression significantly inhibited DLBCL cell viablity in a time-dependent manner (Fig. 2C).

### Overexpression of LINC00963 affects endoplasmic reticulum stress, apoptosis and autophagy

**Fig. 3:**
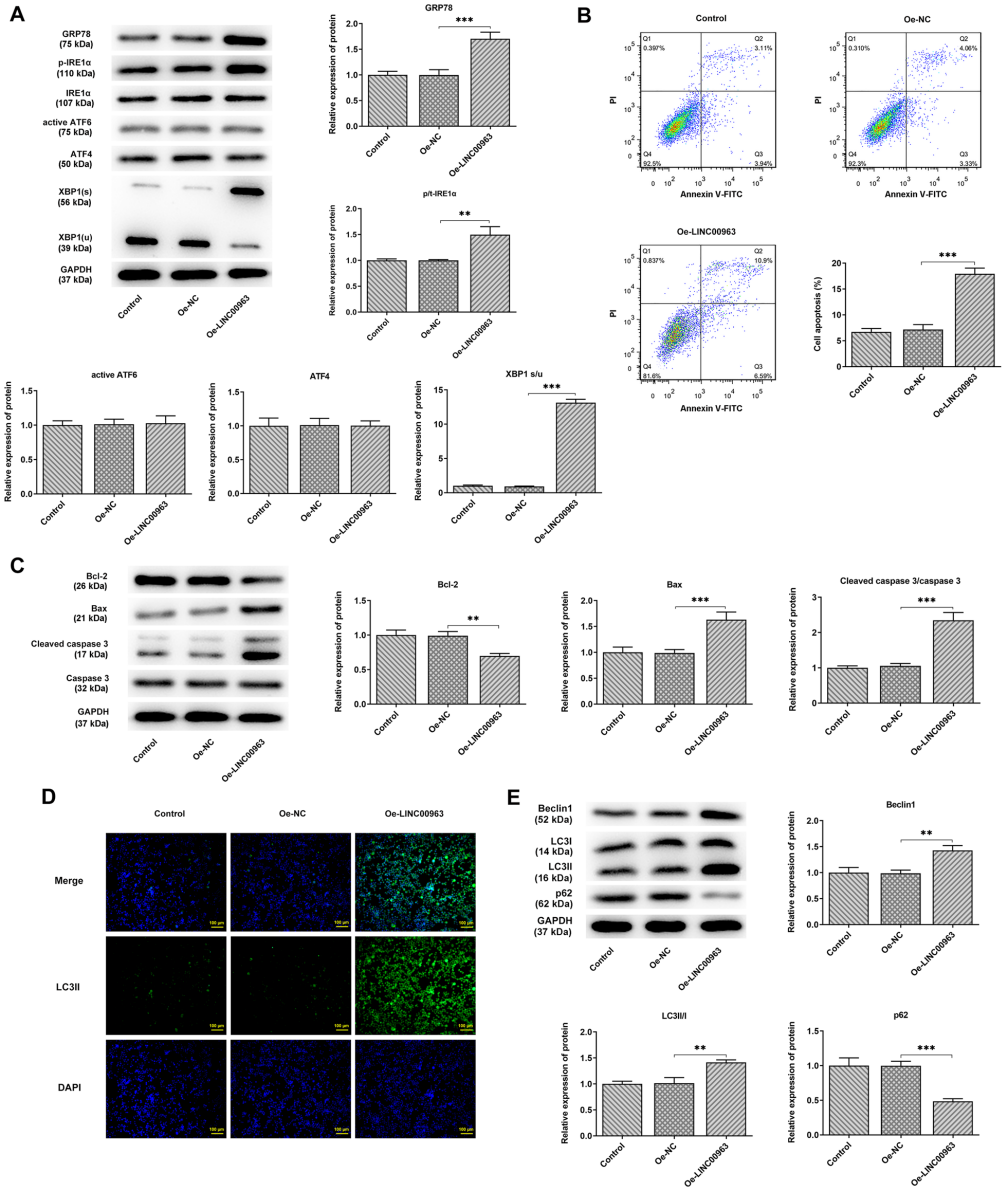
**A** DLBCL cells were analyzed for GRP78, p-IRE1α, IRE1α, active ATF6, ATF4, XBP1(s) and XBP1(u) by Western blot. **B** Flow cytometry was used to detect DLBCL apoptosis after LINC00963 overexpression. **C** LINC00963 overexpression affected apoptosis-related proteins levels. **D** Immunofluorescence analyzed the expression of LC3. **E** The detection of autophagy markers (Beclin1, LC3II, LC3I and p62) through WB

Exogenous overexpression of LINC00963 markedly elevated the protein levels of GRP78 and phosphorylated IRE1α as well as the XBP1(s) to XBP1(u) ratio, but it had no significant impact on the protein levels of IRE1α, ATF6 and ATF4 (Fig. 3A). It can be seen that LINC00963 overexpression mainly activates ER stress-associated IRE1/XBP pathway. It is known that ER stress can induce cell apoptosis. Therefore, the effect of LINC00963 overexpression on the apoptosis of SUDHL4 cells was analyzed subsequently. As demonstrated in Fig. 3B, LINC00963 overexpression group showed more apoptotic cells, increased protein levels of Bax and cleaved caspase3, as well as decreased Bcl-2 expression (Fig. 3C). Previously, the results of GO function enrichment implied that co-expression of LncRNAs could be involved in the regulation of autophagy in DLBCL. To further investigate the function of LINC00963, the expression of LC3 was detected through immunofluorescence assay. The result showed a significant increase in LC3 (green dots) in Oe-LINC00963 group relative to Oe-NC group (Fig. 3D). Western blot further revealed that LINC00963 overexpression promoted the expression of Beclin1 and LC3II while inhibiting p62 expression (Fig. 3E). Furthermore, the protein level of LC3I showed no significant change after LINC00963 overexpression compared to the NC (Fig. 3E). Based on these results, it can be ascertained that LINC00963 overexpression induces autophagy in SUDHL4 cells.

### miR-320a is a direct target of LINC00963

**Fig. 4:**
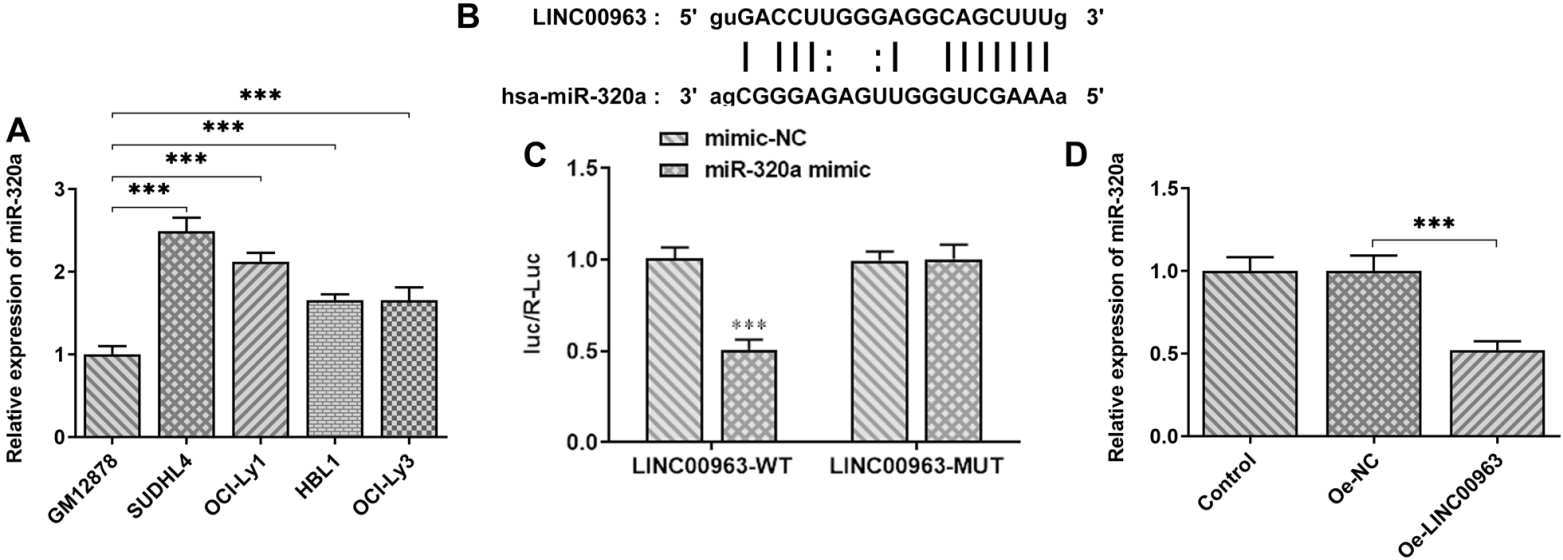
**A** The expression of miR-320a in diffuse large B cell lymphoma cell lines was detected by qPCR. **B** Starbase 3.0 predicted the binding sites of LINC00963 and miR-320a. **C** Luciferase activity was detected. **D** LINC00963 overexpression reduced the expression of miR-320a

The lncRNA-miRNA-mRNA ceRNA network previously revealed the possible existence of a regulatory relationship between LINC00963 and miR-320a in DLBCL. We then investigated whether the effect of miR-320a upregulation on DLBCL cells produced blocked the effects of LINC00963 overexpression. The results of qPCR determined a relatively higher level of miR-320a expression in DLBCL cell lines versus GM12878 cells (Fig. 4A). Bioinformatics analysis showed that LINC00963 contained highly conserved miR-320a binding sites as shown in Fig. 4B. Dual luciferase reporter assay confirmed significantly decreased luciferase activity in the LINC00963 WT (wild type) + miR-320a mimic group, while there was no significant change in the LINC00963 MUT (mutant type) groups (Fig. 4C). qPCR further demonstrated that LINC00963 overexpression significantly reduced the expression of miR-320a in SUDHL4 cells (Fig. 4D), suggesting that LINC00963 may directly target miR-320a in DLBCL.

### XBP1 is the target protein of miR-320a in SUDHL4 cells

**Fig. 5:**
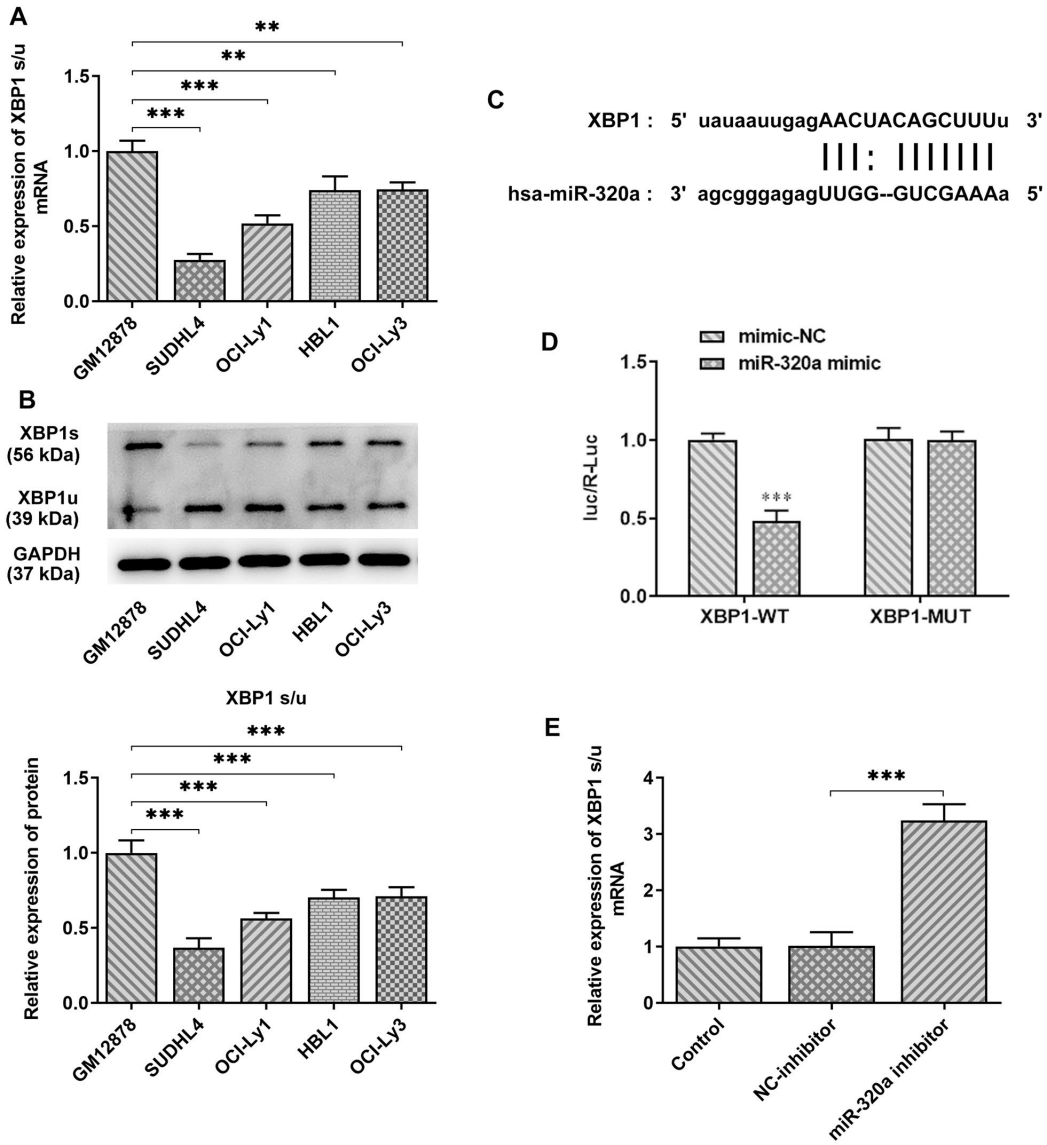
**A, B** The expression of XBP1 in diffuse large B cell lymphoma cell lines was detected by qPCR and Western blot, and the ratio of XBP1s/u was calculated. **C** miR-320a has binding sites with XBP1. **D** Luciferase activity was detected by double luciferase assay after miR-320a mimic was co-transfected with wild or mutant XBP1 3’ UTR. **E** XBP1(u) and XBP1(s) mRNA expression was detected by RT-QPCR, respectively, after transfection of Mir-361-5p inhibitor

The results of bioinformatics analysis indicated that LINC00963/miR-320a could regulate the proteins levels of XBP1. Therefore, the expression of XBP1 was examined in GM12878 and DLBCL cells. A significant reduction in the ratio of XBP1s/u in DLBCL cells was observed by contrast with GM12878 cells. Surprisingly, the ratio of XBP1s/u was found to be lower in SUDHL4 cells than other DLBCL cells (Fig. 5A). Bioinformatics analysis showed that miR-320a shares complementary binding sites with the 3’-UTR of XBP1 mRNA (Fig. 5B). XBP1 protein has two forms, the unspliced form (XBP1u) and the spiced form (XBPls). When cells undergo ER, the endonuclease activity of IRE1 dissociated from glucose-regulated protein 78 (GRP78) is activated through self-dimerization and phosphorylation. Then, IRE1 specifically removes an intron fragment of the sequence of 26 bp in length and finally leads to the production of XBP1s that is involved in ER expansion, protein maturation, misfolded protein degradation and other processes [18, 19]. SUDHL4 cells were co-transfected with XBP1 3’ UTR-WT/Mut and miR-320a mimic/NC for luciferase detection. The results in Fig. 5C, D confirmed the targeting relationship between XBP1 mRNA 3’-UTR and miR-320a. qPCR further verified the relationship between XBP1 and miR-320a, as cells transfected with miR-320a inhibitor showed an obvious increase in the ratio of XBP1 s/u, compared to the NC group (Fig. 5E).

### miR-320a overexpression suppressed LINC00963 overexpression-induced viability, endoplasmic reticulum stress, apoptosis and autophagy

**Fig. 6:**
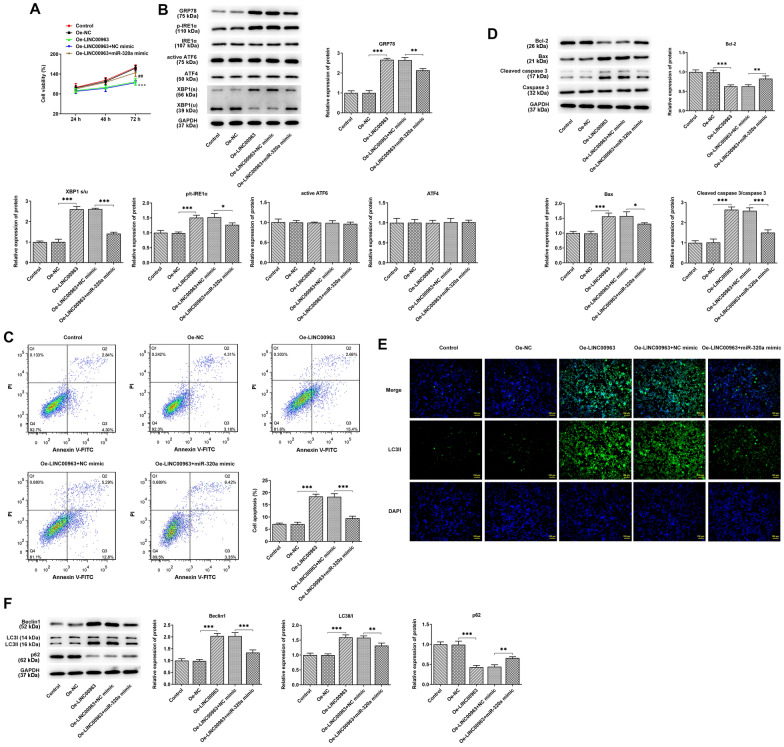
**A** CCK-8 assay was used to detect cell proliferation in different groups. **B** Western Blot was employed to detect the expression of ERs markers, including GRP78, p-IRE1α, IRE1α, active ATF6, ATF4, XBP1(s) and XBP1(u). **C** The analysis of cell apoptosis in each group through Flow cytometry. **D** Western blot analyzed the expression of Bcl-2, Bax, cleaved caspase 3 and caspase 3. **E** The expression of LC3 in SUDHL4 cells was analyzed by Immunofluorescence assay. **F** LINC00963 overexpression regulated the expression of autophagy-related proteins (Beclin1, LC3II, LC3I and p62) through miR-320a

The following experiments were designed to investigate whether LINC00963 affects the viability of SUDHL4 cells through regulating miR-320a. It was found that overexpressing LINC00963 reduced the viablity, whereas co-transfection of Oe-LINC00963 and miR-320a mimic increased cell viablity comparatively (Fig. 6A). In addition, the protein expression levels of GRP78, p-IRE1α, IRE1α, active ATF6, ATF4, XBP1(s) and XBP1(u) were measured in different transfection groups (Fig. 6B). The results showed that GRP78, p-IRE1α and XBP1(s) expressions were significantly increased by LINC00963 overexpression but were further decreased in Oe-LINC00963 + miR-320a mimic group (Fig. 6B). Additionally, the results of flow cytometry showed significantly increased cell apoptosis after transfection of Oe-LINC00963, while co-transfection of Oe-LINC00963 and miR-320a counteracted the effect of LINC00963 overexpression on SUDHL4 cell apoptosis (Fig. 6C). Moreover, the results of western blot showed that LINC00963 overexpression decreased the expression of Bcl-2 and increased that of Bax and cleaved caspase 3, whereas transfection of miR-320a mimic counteracted the effects of LINC00963 overexpression on apoptosis-related protein expression (Fig. 6D). The results suggest that LINC00963 upregulation activates the apoptosis signaling pathway in SUDHL4 cells and induce apoptosis by inhibiting miR-320a expression. Next, the role of miR-320a in LINC00963-regulated autophagy was investigated. Immunofluorescence assay demonstrated a dulled effect of LINC00963 overexpression on promoting the expression of LC3II and the inhibiting that of LC3I after transfection of miR-210a mimic in SUDHL4 cells (Fig. 6E). Furthermore, the protein expression of Beclin1 and LC3II was weakened, and that of p65 was enhanced in the Oe-LINC00963 + miR-320a mimic group in comparison with the Oe-LINC00963 + NC mimic group, indicating that LINC00963 activates autophagy by downregulating miR-320a in SUDHL4 cells (Fig. 6F).

### Overexpression of LINC00963 inhibits the growth of diffuse large B cell lymphoma in vivo

**Fig. 7:**
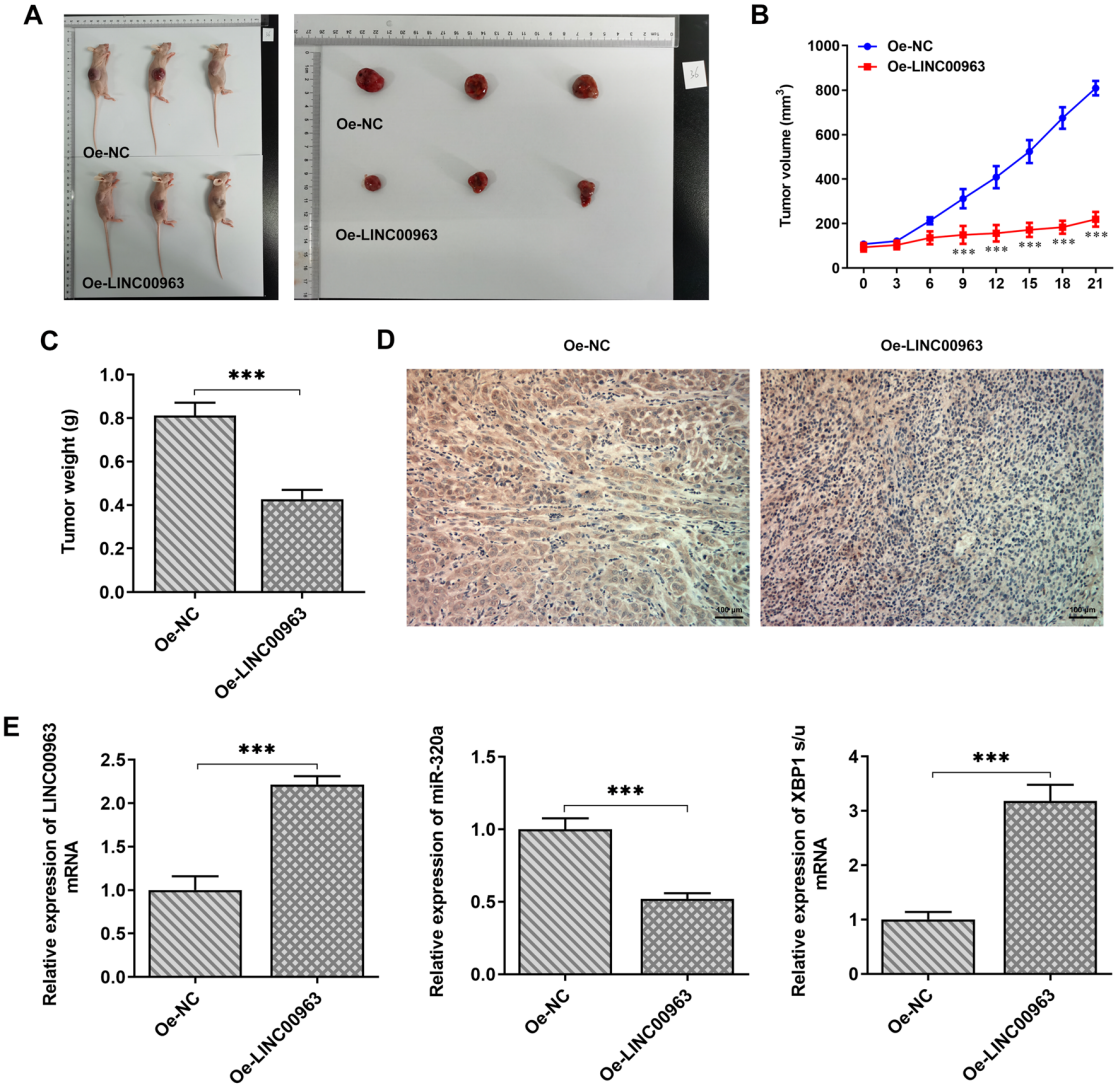
Tumor tissues were taken out, the tumor sizes (**A**) of each group were photographed and measured and the volume (**B**) was calculated, and the volume (**C**) of tumor tissues of each group was statistically analyzed. **D** The expression of Ki67 was detected through immunohistochemical assay. **E** Total RNAs from tumor tissues of each group were extracted for RT-qPCR assay to detect the expression of LINC00963, miR-320a, XBP1(s) and XBP1(u) in each group

Given the regulatory role of LINC00963/miR-320a in ERs, apoposis and autophagy, which could be mediated by XBP1 in vivo, we next wondered to know their roles in vivo. Tumorigenesis was performed in nude mice by subcutaneous injection of SUDHL4 cells. Compared to the Oe-NC group, mice injected with LINC00963-overexpressing SUDHL4 cells exhibited effectively inhibited tumor formation (Fig. 7A) and significantly reduced tumor volume (Fig. 7B) and weight (Fig. 7C). The inhibitory effect of LINC00963 overexpression on tumor growth in vivo was consistent with the results of our in vitro experiments. Ki67, as a tumor cell proliferation marker, was found to present higher expression levels in LIN00473 overexpression group compared to the NC group (Fig. 7D). In addition, LINC00963, miR-320a, XBP1(s) and XBP1(u) were detected by qPCR in the mouse model. The results showed that compared to the NC group, the expression of LINC00963 and XBP(s) was elevated while that of miR-320a and XBP1(u) was decreased in the LINC00473 overexpression group (Fig. 7E).

## Discussion

LINC00963 has been reported to play oncogenic roles in several cancer types including osteosarcoma and breast cancer [16, 20]. In the present study, LINC00963 was found through TCGA data analysis to be expressed at a significantly lower level in DLBCL tissue compared with normal tissue, which was further confirmed by in vitro and in vivo experiments. Mechanistic research on the lncRNA-miRNA-mRNA ceRNA network revealed that LINC00963, miR-320a and XBP1 may collectively form a target network, suggesting that LINC00963/miR-320a/XBP1 could be implicated in the regulation of multiple biological processes in DLBCL. The results of in vitro experiment revealed that the LINC00963/miR-320a axis regulated the processes of ER stress, apoptosis and autophagy in DLBCL cells, while in vivo experiment showed that the progression of DLBCL could be suppressed by the induction of LINC00963 overexpression.

Targeting protein degradation pathways is considered to be a novel approach for the inhibition of lymphoma growth [21]. In the present study, LINC00963 overexpression increased the ratio of XBP1(s)/XBP1(u), the phosphorylation level of IRE1α and the protein level of GRP78 without affecting ATF6 and ATF4 expression, indicating that LINC00963 promoted UPR mainly through regulating IRE1/XBP-1 pathway. Additionally, previous research has established the pro-apoptotic effect of the activation of GRP-78/IRE-1/XBP-1 signaling pathway [22, 23]. In the present study, the result of flow cytometry showed promoted apoptosis of SUDHL4 cells after LINC00963 overexpression, along with a reduction in Bcl-2 expression and an increase in the protein levels of Bax and cleaved caspase 3. IRE1/XBP-1 pathway is involved in mediating cell apoptosis [24]. The involvement of LINC00963 in apoptosis has been confirmed as previously shown in some studies [25, 26]. Based on the present experimental results, LINC00963 could activate the ERs-mediated apoptosis cascade in DLBCL cells via IRE1/XBP-1.

A prognostic value of LINC00963 in DLBCL patients and enriched LINC00963 in autophagy pathway were found through GO function enrichment analysis, which led to the hypothesis that LINC00963 might regulate autophagy in DLBCL. In line with our hypothesis, both in vitro and in vivo DLBCL models showed an increase in the expression of autophagy markers Beclin1 and LC3II and a decrease in that of autophagy receptor p62 after LINC00963 overexpression. Noteworthily, IRE1α-XBP1 pathway has also been reported to be partly responsible for the induction of autophagy in cancer [27, 28].

The oncogenic activity of LINC00963 has been confirmed in some cancers, such as osteosarcoma, breast cancer and ovarian cancer [7, 16, 29]. Recent studies have revealed that LINC00963 not only affects tumorigenesis by functioning as a sponge of miRNAs [30], but regulates the sensitivity of tumor cells to chemotherapy and radiotherapy [16, 31]. Conversely, the present study demonstrated that LINC00963 overexpression accelerated DLBCL cell apoptosis in vitro and inhibited tumor growth in vivo, providing a fresh new insight into the function of LINC00963 in cancer progression. Nucleotide-binding oligomerization domain-like receptors family CARD domain-containing 5 (NLRC5) signaling was reported to participate in B-cell lymphomagenesis [32], which provides a perspective for further studying the mechanism of LINC00963 that there may exist an association between NLRC5 and LINC00963. Luciferase reporter and qPCR assays together proved that LINC00963 sponged the 3’UTR of miR-320a to downregulate miR-320a expression. Additionally, miR-320a overexpression rescued LINC00963 overexpression-promoted UPR, apoptosis and autophagy in SUDHL4 cells. These findings demonstrated that LINC00963 could directly regulate miR-320a to affect the aforementioned biological processes of DLBCL cells. Moreover, a miRNA-mRNA interaction among miR-320a and XBP1 mRNA was validated in this study, and miR-320a inhibitor was found to elevate the ratio of XBP1(s)/XBP1(u) in DLBCL cells. Raman-enhanced spectroscopy (RESpect) probe is the instrument revealing the distinct differences/similarities of potential tumor markers to determine potential malignancies in infants, children and adolescents [33]. The recognition of LINC00963/miR-320a axis provides potential tumor markers for the application of RESpect probe.

Taken together, data in this study illustrated the miR-320a-dependent promoting effect of LINC00963 on the apoptosis and autophagy of SUDHL4 cells. This study also elucidated the regulatory network of LINC00963/miR-320a/XBP1 and its biological function in tumor development, suggesting that both LINC00963 and miR-320a could be potential therapeutic targets for the molecular targeted therapy of DLBCL.

## Conclusions

In the present study, the LINC00963/miR-320a was identified to involve in ERs, apoptosis and autophagy in DLBCL according to in vitro and vivo experimental methods, this role of which was closely associated with XBP1. Lacking of further experimental basis to determine the mechanism in which XBP1-mediated ERs is involved in autophagy and whether targeting LINC00963/miR-320a/XBP1 exerts efficiency in clinic. With regard to this result, the pathological mechanism of DLBCL gets further addition and explanation, which provides basis for developing new markers and targeting medicine for the diagnosis and therapy of DLBCL, respectively.

## Supplementary Information

**Additional file 1.** The original protein bands.

## Data Availability

The datasets used and/or analyzed during the current study are available from the corresponding author on reasonable request.
