# Supplementary figures and images for: The regulation of miR-320a/XBP1 axis through LINC00963 for endoplasmic reticulum stress and autophagy in diffuse large B-cell lymphoma
Source: Cancer Cell Int. 2021 Jun 10;21:305. doi: 10.1186/s12935-021-01992-y (PMC8194177; doi:10.1186/s12935-021-01992-y)

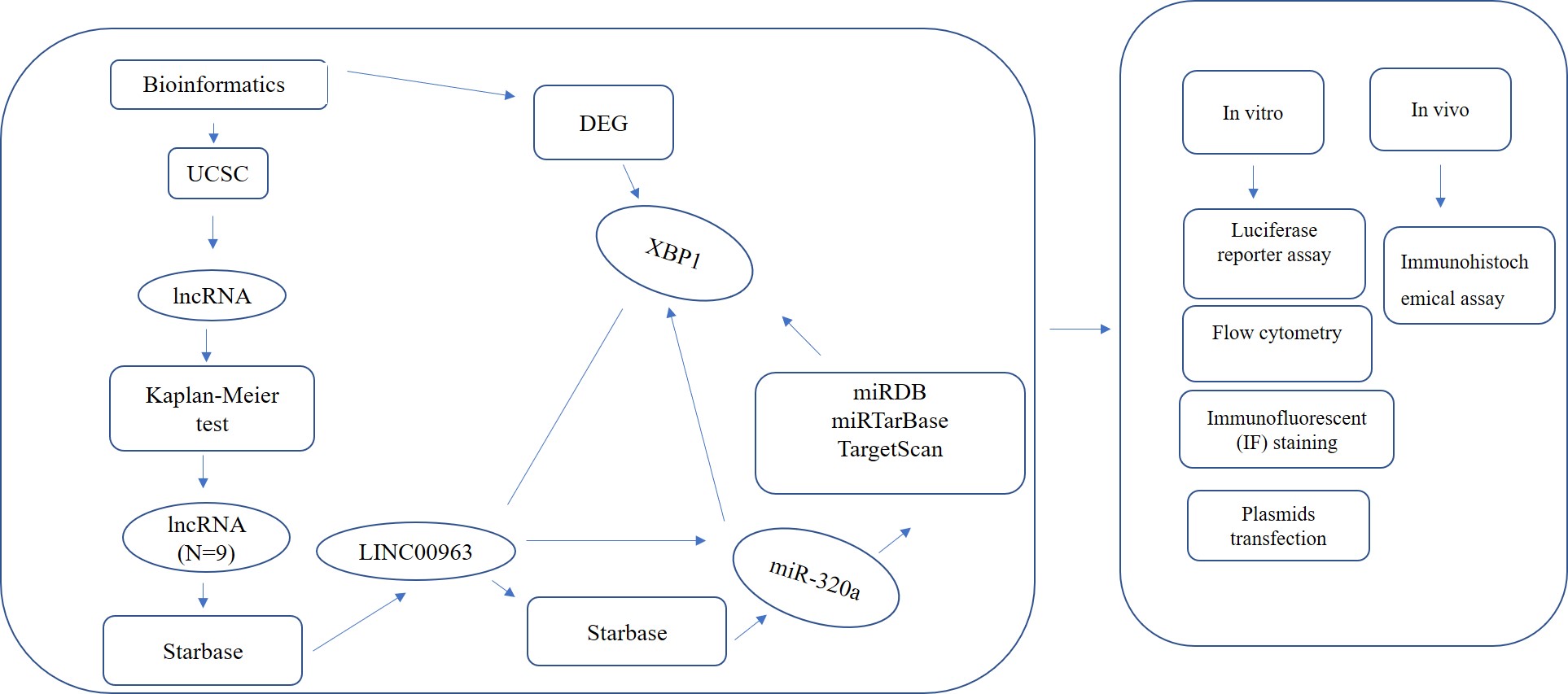

Supplement: Supplementary file 1 — Additional file 1. The original protein bands. [file 12935_2021_1992_MOESM1_ESM.jpg]
